# Surgical outcomes of colorectal cancer surgery in transplant recipients: A matched case–control study

**DOI:** 10.1111/codi.70133

**Published:** 2025-05-30

**Authors:** Phil Meister, Samira Vestweber, Jan Neuhaus, Marc A. Reschke, Ulf Neumann, Andreas D. Rink

**Affiliations:** ^1^ Department of General, Visceral, Vascular and Transplantation Surgery University Hospital Essen Essen Germany

**Keywords:** colorectal cancer, immunosuppressant, surgery, transplantation

## Abstract

**Aim:**

The incidence of colorectal carcinoma (CRC) in transplant (TX) recipients is higher than in the general population. Registry data indicate inferior oncological outcomes for this population. While the general surgical risk is increased in TX recipients, the risk associated with elective CRC surgery in this population is not well investigated.

**Methods:**

TX recipients, who underwent elective surgical treatment for CRC at our specialized centre from 2008 to 2024 were included in this case–control study. The controls were randomly selected from our CRC database and matched according to tumour location and Charlson Comorbidity Index. Outcomes assessed included intensive care unit stay, in‐hospital mortality, length of hospital stay and major morbidity (defined as Clavien–Dindo Grade ≥3).

**Results:**

The study included 24 TX recipients. Ten patients had had either liver or kidney TX, three patients had undergone lung TX and one patient heart TX. The mean time interval between transplantation surgery and CRC was 8.82 years. Morbidity was significantly higher in the TX group (54.2% vs. 8.3%, *P* = 0.001; OR 13.0, 95% CI 2.5–68,1, *P* = 0.002) and length of hospital stay was significantly longer (25 vs. 9 days, *P* = 0.001; OR 9.09, 95% CI 1.4–16.7, *P* = 0.022) for TX patients. No significant differences in mortality and intensive care unit stay were observed.

**Conclusions:**

The risk of surgery for CRC in TX patients is significantly increased. Treatment decisions should involve TX experts to develop a tailored and considered treatment plan.

**What does this paper add to the literature?**

This paper reports the largest case‐matched cohort of colorectal cancer surgery in transplant recipients. The risk for surgery is significantly increased and should be considered for an individualized treatment decision.

## INTRODUCTION

Managing carcinoma in transplant (TX) patients presents significant challenges for both surgical and oncological teams. An increased cancer risk after organ TX has now been thoroughly described in the literature [1, 2, 3]. The increased incidence of colorectal cancer (CRC) had been a subject of extensive discussion for a considerable period [4, 5, 6], and TX was subsequently identified as an independent risk factor for gastrointestinal malignancies [1, 7, 8]. Data regarding the outcome of CRC in TX patients are limited. Merchea et al. reported on a cohort of 20 patients with CRC in TX patients at the Mayo Clinic, demonstrating a short‐term morbidity of 55% and 5‐year survival of 69% at a median follow‐up of 2.4 years [9]. Kim et al. reported on 17 CRC patients after kidney TX, which demonstrated poorer oncological outcome compared to a matched none‐TX control group [10]. More recent studies in subsequent larger cohorts of 63 and 98 patients, respectively, with CRC after TX also confirmed an inferior prognosis after TX, particularly in advanced cancer stages [11, 12]. The latter analysis from the Swedish national database revealed TX patients as less likely to receive adequate abdominal surgery and adjuvant therapy than none‐TX controls [12]. The surgical risks associated with CRC surgery in TX patients remain unclear. Overall operative risk is elevated in this population [13, 14], and colectomy performed for diverticulitis have been shown to exhibit increased morbidity, mortality and length of hospital stay (LOS) [15], particularly in emergency settings [16]. A discussion concerning the adjustment of treatment recommendations for surgical therapy of diverticulitis in TX patients has been initiated [17, 18].

We aim to analyse the risks of CRC surgery in TX patients in our large TX and oncological centre in Germany. Based on our findings, we discuss the need for adapting the management of CRC in TX recipients.

## METHODS

Data were extracted from the digital hospital information system for all patients diagnosed with CRC after TX who underwent surgery at the University Hospital of Essen, Germany, between January 2008 and October 2024. This retrospective study was approved by the local ethics committee (24‐12246‐BO) and followed the Declaration of Helsinki.

Patient characteristics including gender, age, reason and date of TX, immunosuppression and comorbidities were recorded. Surgical procedures were categorized as right‐sided, left‐sided or rectum resection. Outcome was assessed by intensive care unit (ICU) stay, in‐hospital mortality, LOS and morbidity (Clavien–Dindo ≥3). A case match analysis was conducted using patients from our CRC database. Matching was based on tumour location, corresponding surgical procedure and Charlson Comorbidity Index (CCI) (±1). The CCI incorporates age.

### Statistics

Data were analysed using SPSS 29.0 software (IBM, Armonk, NY, USA). A two‐sided *t* test was performed to compare mean values and the 95% CIs were calculated. Data are presented as mean values with standard deviation or median and range as appropriate. Binary logistic regression analysis was used to determine risk factors and other dependences. A *P* value <0.05 was considered statistically significant.

## RESULTS

### Patient characteristics

Twenty‐four patients received surgery for CRC after TX in the stated period. Twelve patients had right‐side and eight left‐side colon cancer, while four patients were treated for rectal cancer. Nineteen patients had localized tumours (Union for International Cancer Control [UICC] I + II), and six patients advanced stages (UICC III + IV). An equally sized control group was randomly matched according to CCI and tumour localization from our CRC database; mean age in the control group was 65.9 years. In the control group 13 patients had UICC I + II classification and 11 patients UICC III + IV (Table 1).

**TABLE 1:** Patient characteristics for transplant patients and their correlating case match control.

	Overall, *n* = 48	Transplant patients, *n* = 24	Control, *n* = 24
Male	26 (54.2%)	11 (45.8%)	15 (62.5%)
Female	22 (48.8%)	13 (54.2)	9 (37.5%)
Age (mean ± SD)	67.2 (±10.7)	68.5 (±9.2)	65.9 (±12.2)
CCI (mean ± SD)	7.5 (±2.5)	7.5 (±2.4)	7.5 (±2.5)
Tumour location			
Right‐side CRC	24 (50%)	12 (50%)	12 (50%)
Left‐side CRC	16 (33.3%)	8 (33.3%)	8 (33.3%)
Rectum	8 (16.6%)	4 (16.6%)	4 (16.6%)
Tumour stage			
UICC I	9 (18.8%)	5 (20.8%)	4 (16.7%)
UICC II	23 (47.9%)	14 (58.3%)	9 (37.5%)
UICC III	6 (12.5%)	2 (8.3%)	4 (16.7%)
UICC IV	10 (20.8%)	3 (6.3%)	7 (29.2%)

Abbreviations: CCI, Charlson Comorbidity Index; CRC, colorectal carcinoma; UICC, Union for International Cancer Control.

### Transplant characteristics

Ten patients had had liver and 10 patients kidney TX, three patients had received lung TX and one patient had had heart TX. The mean time interval between transplantation and surgery for CRC was 8.82 (0.2–30.2) years. The most frequently used immunosuppressive drug was tacrolimus in 70% of patients, 58.3% of the patients were on steroids, 25% had mycophenolatmofetil and 12.5% ciclosporin (Table 2).

**TABLE 2:** Patient characteristics regarding their transplantation and immunosuppression.

	*n* = 24
Liver TX	10 (41.7%)
Lung TX	3 (12.5%)
Kidney TX	10 (41.7%)
Heart TX	1 (4.2%)
Time from transplant to CRC (mean ± SD)	8.82 (min. 0.2– max. 30.2), SD ± 8.08 years
Tacrolimus	17 (70.8%)
Ciclosporin	3 (12.5%)
MMF	6 (25%)
Steroids	14 (58.3%)

*Note*: Patients often received more than one immunosuppressive drug.

Abbreviations: CRC, colorectal carcinoma; MMF, mycophenolatmofetil; TX, transplant.

### Outcome

**FIGURE 1:**
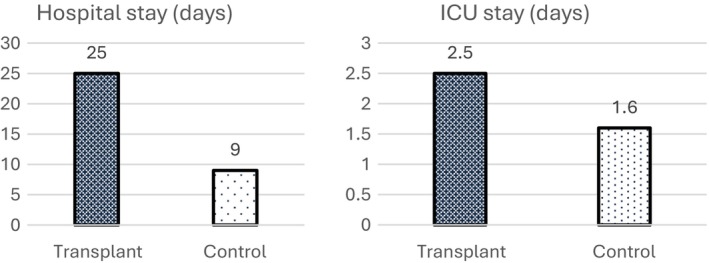
Hospital stay and ICU stay for TX and their case matches after CRC surgery. Two‐sided test for hospital stay *P* = 0.001 and for ICU stay *P* = 0.266.

**FIGURE 2:**
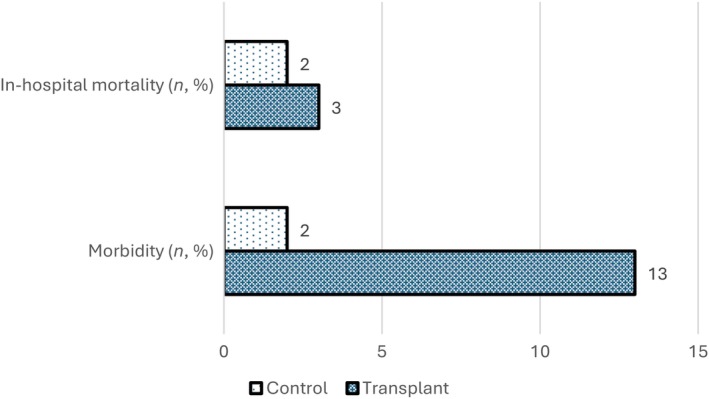
In‐hospital mortality and morbidity (Clavien–Dindo ≥3) for TX patients and their case matches after CRC surgery. Two‐sided *t* test for mortality *P* = 0.32 and for morbidity *P* = 0.001.

Mean LOS in TX patients was 25 days in the TX cohort and 9 days in the control group (*P* = 0.001). ICU stay was longer in the TX group without statistical significance (2.5 days vs. 1.6 days, *P* = 0.26) (Figure 1). Mortality was also increased in TX patients (12.5% vs. 8.3%, *P* = 0.323) but below the statistical significance level. 54.2% of TX patients had major complications (Clavien–Dindo ≥3) after colorectal resection in contrast to 8.3% in the control group (*P* = 0.001) (Table 3, Figure 2). In the TX group five patients received revision surgery (20.8%) and anastomotic leakage occurred in two patients (8%). Complications are listed in Table 4. A comparison of the morbidity rate according to the TX organ did not demonstrate significant differences: kidney TX patients had longer LOS than liver TX patients (35.7 days vs. 20.1 days, *P* = 0.2), liver TX had longer ICU stay than kidney TX (3.8 days vs. 1.9 days, *P* = 0.5), and no differences in the mortality rate were present. Comparison of patients with CRC less than 5 years after TX with patients long‐term post TX (>5 years) showed no differences in the relevant endpoints.

**TABLE 3:** Outcome of colorectal cancer surgery.

	Overall	Transplant	Control	*P*
Length of hospital stay (days, mean ± SD)	17 (±19.4)	25 (±25.3)	9 (±3.7)	<0.001
ICU stay (days, mean ± SD)	2 (±4.8)	2.5 (±5.4)	1.6 (±4.1)	0.266
Morbidity (*n*, %)	15 (31.3%)	13 (54.2%)	2 (8.3%)	<0.001
In‐hospital mortality (*n*, %)	5 (10.4%)	3 (12.5%)	2 (8.3%)	0.323

*Note*: *P* values via two‐sided *t* test.

Abbreviation: ICU, intensive care unit.

**TABLE 4:** Overview of morbidity and outcome listed for each patient.

Transplant/control	Side	CCI	Complication	Hospital stay	Outcome
TX	Right	12	Cardiogenic shock	Death on day 3	In‐hospital death
TX	Left	6	Surgical wound revision	46 days	Discharge
TX	Left	6	Bleeding	10 days	Discharge
TX	Right	9	Ileus, surgical revision	10 days	Discharge
TX	Rectum	5	Anastomotic leak	101 days	Discharge
TX	Right	10	Sepsis, renal failure	59 days	Discharge
TX	Rectum	9	Ileus, surgical revision	29 days	Discharge
TX	Right	6	Ileus, aspiration, sepsis	Death on day 7	In‐hospital death
TX	Right	11	Bleeding	19 days	Discharge
TX	Left	11	Ileus, aspiration, sepsis	Death on day 6	In‐hospital death
TX	Left	10	Anastomotic leak	56 days	Discharge
TX	Right	6	Ileus, surgical revision	64 days	Discharge
Control	Right	10	Ileus, sepsis	Death on day 25	In‐hospital death
Control	Left	12	Sepsis, ACLF	Death on day 5	In‐hospital death

Abbreviations: ACLF, acute on chronic liver failure, cirrhosis patient; CCI, Charlson Comorbidity Index; TX, transplant.

### Regression

Univariate regression demonstrated a substantial increase in the odds of complications for TX patients compared to controls (OR 13.0, 95% CI 2.5–68.1, *P* = 0.002). Additionally, TX status was significantly associated with an increased LOS, with an OR of 9.09 (95% CI 1.4–16.7, *P* = 0.022). However, no significant difference in in‐hospital mortality was observed between TX and control patients (OR 1.57, 95% CI 0.23–10.36, *P* = 0.63). Other factors examined in univariate regression, including the CCI (OR 2.05, 95% CI 0.72–5.82, *P* = 0.17), time elapsed since TX (OR 1.23, 95% CI 0.88–1.73, *P* = 0.212) and rectal versus right‐sided tumour location (OR 16.7, 95% CI 0.12–2271, *P* = 0.26) did not demonstrate statistically significant associations with complications.

## DISCUSSION

Managing CRC in TX patients presents a significant challenge for clinicians. The incidence of CRC is elevated in this population [1, 2] and numerous studies have reported significantly poorer survival outcomes [12, 19]. Only one Korean cohort has reported comparable survival outcomes in 66 CRC patients following TX [8]. Limited knowledge exists regarding the management of CRC in this population due to the relatively small number of reported cases. The aforementioned Korean study, which included 66 patients, reported CRC incidence of less than 1% in a database of approximately 8000 TX recipients. Data regarding surgical risk in this population are particularly scarce. Khoury and coworkers analysed a cohort of 55 immunocompromised patients with CRC, showing no increased perioperative risk but worse long‐term oncological outcome [20]. Conversely, numerous studies have reported an increased risk for colectomy in immunocompromised or transplanted patients due to diverticulitis [15, 16, 21, 22]. The reported rate of major complications (Clavien–Dindo ≥3) exceeded 50%, aligning with our findings. Previous studies have generally demonstrated an increased operative risk for abdominal surgery in kidney TX patients [13, 14].

Limitations of this study include the relatively small sample size, which precluded a definitive assessment of possible differences in mortality and ICU stay. Due to the low incidence of CRC in this population recruiting larger cohorts is challenging. Even nationwide registry studies have reported fewer than 100 patients [12]. Still, to the best of our knowledge, this study represents the largest single‐centre case–control study for CRC surgery in TX recipients.

The control group was adequately matched for CCI and tumour location according to the study design. Consequently, age was also matched. Differences were observed in UICC stage, with more advanced cases noted in the control group. However, the UICC staging system is not especially designed to predict perioperative risk, as tumour location influences biology and metastatic patterns, thereby impacting UICC stage [23, 24]. The observed increase in morbidity and LOS appears to be a reliable finding. LOS of 9 days in the control group appears long under consideration of modern enhanced recovery after surgery concepts [25]. This can be explained by the high CCI in both groups, which means that even the control group consists of morbid and challenging patients.

The relevant mortality in both groups can also be explained by the selection of high‐risk patients. In a recently published study on the prognostic value of the CCI on LOS, morbidity and mortality of surgery for CRC Zhang et al. found that a CCI ≥3 was already associated with highly increased risk of in‐hospital mortality (OR 16.83, 95% CI 2.23–126.88, *P* = 0.0062) [26]. The mean CCI in our cohort was 7.5, indicating a group of patients at extreme risk of complications.

Decision making for CRC in TX patients is complex. The increased operative risk should be carefully considered, potentially necessitating a re‐evaluation of current treatment guidelines. However, it is important to note that chemotherapy in TX patients also presents unique challenges [27]. These challenges in surgical and oncological management may contribute to suboptimal treatment in some TXs, as suggested by current data [12]. Given that surgery remains the only curative option for CRC and no significant increase in perioperative mortality has been consistently demonstrated, these challenges should not preclude patients from receiving appropriate treatment. The inclusion of TX experts in the tumour boards and performing surgery at centres with specialized expertise in TX oncology may improve decision making [12, 28]. At this stage, other specific adjustments, such as switching from traditional immunosuppressants to mTOR inhibitors should be considered [28]. The need for more frequent colonoscopy screening in TX recipients has been a subject of ongoing debate for years [4, 29]. While the incidence of CRC is relatively low, the aggressive tumour biology, particularly in younger patients [30], underscores the importance of early detection, especially given the poorer outcomes observed in advanced stages [12]. Prospective studies are needed to validate the use of intensified colonoscopy screening in TX patients.

## CONCLUSIONS

Surgical risk for CRC in TX recipients is significantly elevated. Our study is the largest case match series in this population. In TX recipients morbidity exceeds 50% and the LOS is notably longer, with a mean stay of 26 days. In our series this did not result in increased mortality. Treatment should involve a multidisciplinary team, including TX experts, and should be performed in centres specializing in both colorectal and TX surgery.

## AUTHOR CONTRIBUTIONS

**Phil Meister:** Conceptualization; investigation; writing – original draft; writing – review and editing; visualization; methodology. **Samira Vestweber:** Investigation; writing – original draft; resources; writing – review and editing. **Jan Neuhaus:** Writing – review and editing; investigation. **Marc A. Reschke:** Writing – review and editing; validation. **Ulf Neumann:** Resources; supervision; writing – review and editing; project administration. **Andreas D. Rink:** Writing – review and editing; writing – original draft; conceptualization; methodology; validation; supervision; project administration.

## FUNDING INFORMATION

No external funding was acquired for this study.

## CONFLICT OF INTEREST STATEMENT

All authors declare that they have no potential conflict of interest concerning this study.

## ETHICS STATEMENT

This study was approved by the local ethics committee (Medical Faculty, University of Duisburg‐Essen, 24‐12246‐BO) and followed the Declaration of Helsinki. No additional patient consent was required for retrospective analysis. The manuscript is in accordance with the STROBE statement.

## Data Availability

The datasets analysed during the current study are not publicly available due to patient privacy limitations but are available from the corresponding author on reasonable request.
